# The Impact of Chemotherapy, Radiation and Epigenetic Modifiers in Cancer Cell Expression of Immune Inhibitory and Stimulatory Molecules and Anti-Tumor Efficacy

**DOI:** 10.3390/vaccines4040043

**Published:** 2016-11-14

**Authors:** Jessica Ann Chacon, Keith Schutsky, Daniel J. Powell

**Affiliations:** 1 Ovarian Cancer Research Center, Department of Obstetrics and Gynecology, Perelman School of Medicine, University of Pennsylvania, Philadelphia, PA 19104, USA; jchacon@mail.med.upenn.edu (J.A.C.); kschutsk@mail.med.upenn.edu (K.S.); 2 Center for Cellular Immunotherapies, Perelman School of Medicine, University of Pennsylvania, Philadelphia, PA 19104, USA; 3 Department of Pathology and Laboratory Medicine, Abramson Cancer Center, Perelman School of Medicine, University of Pennsylvania, Philadelphia, PA 19104, USA

**Keywords:** DNA destabilizers, chemotherapy, radiation, histone deacetylase inhibitor, PD-L1, CTLA-4

## Abstract

Genomic destabilizers, such as radiation and chemotherapy, and epigenetic modifiers are used for the treatment of cancer due to their apoptotic effects on the aberrant cells. However, these therapies may also induce widespread changes within the immune system and cancer cells, which may enable tumors to avoid immune surveillance and escape from host anti-tumor immunity. Genomic destabilizers can induce immunogenic death of tumor cells, but also induce upregulation of immune inhibitory ligands on drug-resistant cells, resulting in tumor progression. While administration of immunomodulatory antibodies that block the interactions between inhibitory receptors on immune cells and their ligands on tumor cells can mediate cancer regression in a subset of treated patients, it is crucial to understand how genomic destabilizers alter the immune system and malignant cells, including which inhibitory molecules, receptors and/or ligands are upregulated in response to genotoxic stress. Knowledge gained in this area will aid in the rational design of trials that combine genomic destabilizers, epigenetic modifiers and immunotherapeutic agents that may be synergized to improve clinical responses and prevent tumor escape from the immune system. Our review article describes the impact genomic destabilizers, such as radiation and chemotherapy, and epigenetic modifiers have on anti-tumor immunity and the tumor microenvironment. Although genomic destabilizers cause DNA damage on cancer cells, these therapies can also have diverse effects on the immune system, promote immunogenic cell death or survival and alter the cancer cell expression of immune inhibitor molecules.

## 1. Introduction

Primary and recurrent solid cancers are often characterized by the intratumoral presence of various immune cells, particularly T lymphocytes, B cells, NK cells, macrophages and other antigen presenting cells. Accumulation of CD3^+^ Tumor-Infiltrating Lymphocytes (TILs) is a favorable prognostic indicator in most solid cancers. Specifically, the presence of cytotoxic CD8^+^ TILs is highly prognostic for survival, indicating a functional role for these cells in the control of cancer progression. This also suggests that therapeutic agents that concomitantly kill cancer cells and induce or bolster host anti-tumor immunity will improve patient outcome [1,2,3,4,5,6,7]. Thus, one major factor that may determine the success or failure of anti-cancer agents is whether they sufficiently engage and stimulate the immune system to induce potent anti-tumor effects. The three-stage model of cancer immunoediting and immunosurveillance proposed by Schreiber and others highlights the importance of the interaction between the immune system and the evolving cancer cells [8,9]. In the “elimination” stage of cancer immunoediting, immunogenic tumor cells are destroyed, while tumor cells that exhibit less immunogenic properties may persist. At a subsequent “equilibrium” stage, cancer cells and the immune system are in balance, and tumor cells are thus stagnant. However, these cancer cells can acquire various “escape” mechanisms, including modes of immunosuppression, that allow the cancer cell to evade the immune system’s methods of recognition and destruction and proliferate. Bearing this theory in mind and considering the growing promise of immunotherapeutic approaches for cancer treatment, there is now great interest in identifying commonly-administered clinical agents, such as genome destabilizers, that are both cytotoxic to cancer cells and promote a cancer cell “elimination” through concurrent induction of immunogenic cancer cell death and inhibition of immune evasion mechanisms. Although genome destabilizers, such as chemotherapeutics and irradiation, are traditionally regarded as immune-depleting [10], it is increasingly evident that conventional chemotherapies, as well as novel epigenetic modifiers and targeted anti-cancer agents, possess both immune-potentiating mechanisms of action, which can enhance immune-based cancer destruction, as well as immune suppressing mechanisms that promote tumor cell growth.

Our review covers the following steps of the “cancer-immunity” cycle as elegantly described by Chen and Mellman [11]: Step 1: release of cancer cell antigens through Immunogenic Cell Death (ICD); Step 2: cancer antigen presentation (release of cytokines, release of ATP, exposure of HMGB1/CRT, TLR engagement); Step 3: priming, activation or suppression T cells (CD28, CD137, CD27, CTLA-4 and PD-L1); and Steps 6–7: T cell recognition and tumor elimination major histocompatibility complex (MHC) and inhibitory ligand expression on tumors, leading to a potentiated or suppressed CTL response). 

Understanding how particular DNA destabilizers affect the expression of immunostimulatory and immunosuppressive ligands and their receptors, alter antigen-presentation or induce immunogenic cell death will greatly impact the success of novel adjunctive therapies. In this review, we discuss the specific roles that conventional and non-conventional genomic destabilizers have on anti-tumor immunity and on inducing immune inhibitory or stimulatory molecules on cancer cells and how they may be best applied to promote the cancer cell elimination.

## 2. Impact of Chemotherapy on Anti-Tumor Immunity and Cancer Cell Immunogenicity

**Figure 1:**
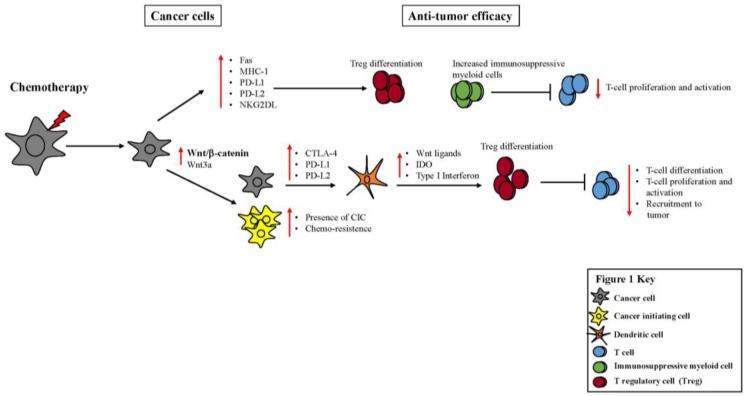
Impact of chemotherapy on cancer cells and anti-tumor efficacy. Chemotherapy elicits cell death of cancer cells. However, chemotherapy can also upregulate inhibitory ligands and pathways, such as PD-L1/2, CTLA-4 and Wnt/β-catenin, as well as promote the differentiation of suppressive cells, such as T regulatory cells and myeloid cells, prompting the suppression of the immune system, ultimately leading to loss of tumor control.

Our first section describes the impact chemotherapy has on anti-tumor immunity, as well as its effect on the expression of inhibitory checkpoint molecules, including PD-L1 (Figure 1). In addition, we will discuss the influence the Wnt/β-catenin pathway has on inducing chemo-resistance in cancer cells (Figure 1).

### 2.1. Chemotherapy Induces Immunogenic Cell Death and Potentiates the Immune Response through Upregulation of MHC, Tumor-Associated Antigens and Co-Stimulatory Ligands on Immune Cells

Chemotherapeutic agents directly induce DNA damage and cancer cell death via immunogenic cell death, apoptosis and other forms of non-apoptotic death, including senescence, mitotic catastrophe and autophagy. For example, a large number of chemopreventative agents promote immune system recognition and elimination of malignant cells in a process called ICD [12,13,14]. This process differs from conventional apoptosis, which can be considered immunogenically silent (exerting no impact on the immune system) or tolerogenic (promoting self-tolerance by dampening immune influences) [15,16]. In ICD, an immune response is elicited against dead cell antigens involving epitope recognition by antigen-presenting cells, Dendritic Cell (DC) activation, MHC presentation, priming of T lymphocytes and cytokine release [17] (Table 1). In ICD, DCs that engulf dying cells receive maturation signals from toll-like receptors (i.e., TLR4) and cytokines, preventing the development of immune tolerance by the activation of specific T cell subsets [18,19,20]. A strict definition of immunogenic cell death typically includes chemopreventative agents, which induce (a) endoplasmic reticulum stress, (b) autophagy, (c) Calreticulin (CRT) translocation, (d) ATP/HMGB1 release and (e) Type I IFN production. Binding of CRT, ATP and HMGB1 to CD91, P2RX7 and TLR4, for example, promotes DC infiltration, engulfment of dying cell antigens and MHC-dependent activation of T cells. In short, this sequence of events causes the release of pro-inflammatory cytokines (IL-1β, IL-17, IFN-γ), promoting the activation, proliferation, and/or infiltration of cytotoxic T lymphocytes, γδ T cells and other immune cells that collectively mediate the destruction of tumor cells [15]. The anthracyclines doxorubicin, epirubicin, idarubicin and mitoxantrone induce ICD in breast adenocarcinoma, fibroblastic sarcoma and colon carcinoma animal models (Table 1) [21,22,23,24,25]. The protective immune response they elicit in vivo is associated with the induction of ER stress, autophagy, CRT/ERp57 translocation, ATP release, CD8^+^ T cell infiltration, pro-inflammatory cytokine secretion and secondary necrosis [26,27,28,29,30,31,32]. In lymphoma and glioma mouse models, cyclophosphamide facilitates ICD through processes involving CRT translocation, HMGB1 release and activation of cytotoxic T lymphocytes, NK cells, macrophages and other immune cells [33,34]. Similarly, administration of the proteasome inhibitor, bortezomib, resulted in immunogenic cell death in lymphoma and myeloma xenografts in mice through CRT redistribution and upregulation of heat shock proteins (HSP70/90), facilitating DC maturation and activation [35,36,37,38]. Likewise, the DNA-damaging agent, bleomycin, triggers ICD in colon carcinoma via ER stress, autophagy, redistribution of CRT/ERp57, ATP secretion, HMGB1 release, CD8^+^ T lymphocyte activation and IFNγ production [24,39,40]. In the same vein, oxaliplatin (but not cisplatin) induces ICD in colon cancer and mouse sarcoma via processes involving TLR4 activation, ER stress, autophagy, CRT translocation and HMGB1 release [26,41,42].

In addition to the initiation of ICD, the ability of conventional chemotherapies to upregulate MHC classes I and II enables malignant cells to be better recognized and destroyed by the immune system [43,44]. Tumor-Associated Antigens (TAAs) are presented on MHC class I to CD8^+^ cytolytic T cells, which can then directly eliminate cancer [45]. Upregulation of HLAs (human leukocyte antigens) corresponding to MHC class II stimulate the proliferation of helper T cells (CD4^+^), which in turn stimulate B cells to produce antibodies against antigens. Recruitment of T helper cells and B cells to the tumor microenvironment primes CD8^+^ T cells and further enhances immune effector functions [46]. Notably, chronic downregulation of HLA in many human cancers impairs CD8^+^ T lymphocyte recognition, limiting the efficacy of immune therapies [47,48]. Accumulating evidence suggests that conventional chemotherapeutics upregulate both MHC class I and II expression in various malignancies through a variety of mechanisms. Anti-cancer agents, such as epothilone B, taxol and vinblastine, which are microtubule destabilizers, induced HLA class I surface protein expression and RNA transcription in a time- and concentration-dependent manner in ovarian cancer cells [49]. Microtubule destabilizers, such as these, potentially alter HLA expression by upregulating the production of cytokines (IFNα, IL-1β, IL-6, IL-12) involved in HLA expression or disrupt intracellular trafficking of HLA proteins, causing redistribution at the tumor surface [49,50]. In our laboratory, administration of type I IFNs (α, β) or type II IFN (γ) dose-dependently increased RNA transcription of HLA class I (A, B, C) and class II (DO, DM) molecules in the vast majority of epithelial cancers examined, while sensitizing HLA-A2^+^ malignancies to antigen-dependent, T cell attack, despite concurrent upregulation of PD-L1/2 [51]. In addition, HLA-B RNA expression is elevated in ovarian cancer patients treated with paclitaxel-carboplatin and exposure of paclitaxel or gemcitabine to murine or human ovarian cancer lines results in a concentration-dependent increase in intracellular and surface MHC class I protein expression in vitro and in vivo [43,52]. Similarly, oxaliplatin, gemcitabine and cyclophosphamide augments HLA (A,B,C) surface protein expression in kidney, breast, prostate and colon tumors in a concentration-dependent manner [23,24,53,54,55] (Table 1). However, not all conventional chemotherapeutics and epigenetic modifiers may induce MHC class I and II expression. For example, we found that carboplatin failed to upregulate MHC class I surface protein expression and RNA transcription in several ovarian cancer cell lines examined [1]. In addition, the Histone Deacetylase Inhibitor (HDACi), depsipeptide FR901228, induces little or no elevation of MHC class I surface protein expression in leukemia, lymphoma, breast and cervical cancer cell lines [56].

In mouse models, pre-conditioning the host using chemotherapy- or radiation-induced lymphodepletion may improve the functionality and survival of transferred T cells in an Adoptive Cell Transfer (ACT) model (Table 1) [57,58]. After patients with colorectal cancer or breast cancer receive traditional chemotherapy, a high-density of Tumor-Infiltrating Lymphocytes (TIL) is often predictive of relapse-free survival [59], rationalizing T cell infusions as a form of therapy. In ACT, lymphodepletion may improve the survival and function of the transferred T cells through multiple mechanisms, including depletion of suppressive T regulatory cells (Tregs), homeostatic proliferative cytokine (IL-7 and IL-15) recruitment and availability, release of immunostimulatory gut microflora and creating homeostatic space for the transferred cells [60,61]. One type of lymphodepleting agent, fludarabine, a purine analog, induces transient lymphopenia with reduced CD4^+^ T cell counts and increases the plasma levels of IL-7 and IL-15 in patients treated with ACT [62]. Although pre-conditioning the host with fludarabine induced the transferred T cells to persist in vivo longer, fludarabine induced lymphodepletion also increased the percentage, but not the absolute number, of CD4^+^ cells that express Foxp3 [62].

Another type of chemotherapy that induces lymphodepletion is the alkylating agent cyclophosphamide. Fludarabine and cyclophosphamide are often used to pre-condition patients receiving adoptive T cell therapy. Interestingly, Ding et al. demonstrated that in mice with lymphoma, pre-conditioning of the host via cyclophosphamide prior to adoptive transfer of antigen-specific CD4^+^ T cells resulted in a robust anti-tumor immune response, but drove the expansion of immunosuppressive monocytic myeloid cells characterized by CD11b^+^, Ly6C^hi^ and CCR2^hi^ [76]. Collectively, it appears that administration of preparative chemotherapeutics may serve to both promote and inhibit T cell responses in the tumor microenvironment and that these effects may be influenced by the type, dose (concentration and schedule) and timing of chemotherapy relative to the administration of immunotherapy. However, the pre-conditioning effects of lymphodepleting chemotherapy in advance of ACT with potent tumor-reactive T cells may outweigh these local effects.

Immune cells that encounter antigen become activated and differentiate, and with chronic antigen stimulation, these cells differentiate to a point of cellular senescence. The differentiation status of immune cells can be determined by the expression of specific markers, such as those associated with co-stimulation, migration and homing, exhaustion and senescence. CD57 is a marker present on a subset of T cells and NK cells that have differentiated to a stage of cellular senescence or have lost the capacity to proliferate in vitro and exhibit a decreased telomere length [77]. While DNA-damaging chemotherapeutic agents induce premature cellular senescence of immune cells, cells expressing CD57 exhibit increased resistance to DNA-damaging chemotherapeutic agents [77], suggesting that intratumoral effector memory T cells may persist long term and mediate control of tumor progression even in patients receiving standard of care chemotherapy. CD28 is a critical co-stimulatory molecule that also serves as a marker of immune cell differentiation. CD28 is expressed on less differentiated cells, but is lost at later stages of differentiation. When the expression of CD28 and CD57 was evaluated, cells that are CD28^−^CD57^+^ are categorized as terminally-differentiated effector memory cells that may display signs of senescence [77]. After one cycle of chemotherapy, there was a significant decrease in the absolute number of naive and memory cells displaying CD28^+^CD57^−^ on their surface [77]. However, the subset expressing CD57 (CD28^+^CD57^+^ and CD28^−^CD57^+^) decreased at a much slower rate [78], indicating that CD57-expressing cells may be more resistant to DNA-damaging chemotherapeutic agents than the CD57^−^ subsets, irrespective of CD28 expression, although a dynamic change in phenotype may account for this observation, as well.

Although chemotherapy can selectively target rapidly-dividing cancerous cells, drug resistance may occur if some cancer cells are not in the synthesis (S) phase of the cell cycle when the chemotherapeutic drug is given. Since the immune system can act upon cancer cells independently of the cancer cell cycle state, the combination of chemotherapy with immunotherapy agents has become a topic of intense investigation. For instance, the pro-inflammatory cytokine IL-18 has been combined with chemotherapeutic agents for the treatment of the murine spontaneously tumorigenic MOSE ovarian cancer cell line ID8 [79]. ID8 represents a cell line derived from spontaneous malignant transformation of C57BL/6 MOSE cells in vitro [79]. IL-18 is a pleiotropic cytokine, originally identified as an IFN-γ inducing factor, that can modulate anti-tumor responses by promoting the expansion of immune cells that express IL-18 receptor α/β chains including CD8^+^ T cells, γδ T cells and NK cells. Although chemotherapeutic agents’, paclitaxel and topotecan, treatment alone results in partial cytotoxicity against the ID8 ovarian cancer cell line, the surviving tumor cells display increased expression of MHC-I and Fas, the cell surface death receptor [79]. MHC-I and Fas induction sensitized the tumor cells to immune cell attack, specifically by CD8^+^ T cells [79]. Based on these findings, a phase I trial was performed of recombinant human IL-18 in combination with PEGylated Liposomal Doxorubicin (PLD), which resulted in a 6% partial objective response rate and a 38% stable disease rate in subjects with recurrent ovarian cancer [80]. More work is required in the area of combination chemotherapy and immunotherapy.

### 2.2. Chemotherapy Induces the Expression of Inhibitory Checkpoint Molecules, NKG2D Ligands and Pathways

In the tumor microenvironment, the interaction between the immune checkpoint receptor PD-1 principally expressed by T cells and its ligands PD-L1 and PD-L2, expressed on the surface of cancer cells and antigen-presenting cells, results in attenuated activation, proliferation and effector functions of T cells and other immune cells, hindering existent immune responses against aberrant cells. At steady state, PD-L1 expression may be clustered in tumor tissues and localized to areas of T cell infiltration where IFN-γ is released, inducing PD-L1 expression [81]. Importantly, administrations of various chemotherapeutics can upregulate PD-L1 (B7-H1) and PD-L2 (B7-DC) expression across a wide range of malignancies. Unfortunately, less is known about the effects of chemotherapy on non-PD-L1 checkpoints, including CTLA-4, PD-L2, Tim-3, lag-9 and galectin-9.

Administration of paclitaxel, etoposide or 5-flurouracil dose- and time-dependently increases PD-L1 surface expression in breast cancer cells, possibly through the JAK/STAT, MAP kinase and/or PI3K/AKT mechanisms, and acts concomitantly with IFNγ to augment the expression of PD-L1 (Table 2) [82]. Similarly, paclitaxel-carboplatin transiently induces PD-L1 surface protein expression on ovarian cancer patient tumors, and paclitaxel, gemcitabine and carboplatin potentiates surface/intracellular protein, as well as RNA transcription of CD274 in human/mouse ovarian cancer cell lines via NFKβ signaling [43,52]. In addition, trabectedin, a chemopreventative agent approved for sarcoma and in clinical trials for ovarian, prostate, breast and pediatric cancers, increased PD-L1 surface and intracellular protein expression in a murine model of ovarian cancer, which was IFNγ-dependent [83].

Paclitaxel also increased PD-L1 surface/intracellular protein and RNA transcription in colorectal and hepatocellular carcinoma through MEK-ERK (MAPK) signaling [84]. Via the same mechanism, cisplatin augmented PD-L1 surface protein expression in liver cancer [85]. In addition, recent results demonstrate that chemotherapeutic agents may alter the expression of specific microRNAs, which, in turn, regulate levels of PD-L1. In retinoblastoma, miR-513a-5p (microRNA) signaling augmented PD-L1 protein and RNA expression after etoposide treatment [86]. Conversely, miR-34a overexpression inhibited upregulation of PD-L1 surface and intracellular protein by arsenic trioxide, a chemopreventative agent used in conjunction with All-Trans Retinoic Acid (ATRA) to treat leukemia [87]. In addition, other miRs (miR-20b, miR-21, miR-130b, Mi570) have been implicated in influencing the expression of PD-L1 in various malignancies [88,89].

Other factors that may contribute to PD-L1 expression on tumor cells are oncogenic driver mutations. For example, lung adenocarcinomas that contain the KRAS mutation may exhibit increased expression of PDL1, compared to wild-type tumors that do not harbor the KRAS mutation [90]. However, oncogenic driver mutations do not always elicit an increase in PD-L1 expression. In melanoma, the presence of the common oncogenic BRAF-V600E mutation did not correlate with increased PDL1 expression [91]. Microsatellite Instability (MSI) tumor conditions may also play a role on the induction of PD-L1 on tumor cells. MSI tumors have been found to express high levels of various immune checkpoint molecules, such as PD-1, PD-L1 and CTLA-4 [92]. In MSI colon tumors, CD4^+^ TIL produces large amounts of IFN-γ, which could then increase the levels of PD-L1 on tumor cells [92]. Adaptive resistance is the term that describes this scenario in which cancer cells utilize augmented PD-1/PD-L1 signaling, with PD-L1 upregulated by IFN-γ secreted by immune cells, to thwart the anti-tumor immune response [93].

Several chemotherapeutic agents upregulate cancer cell expression of NKG2DLs, a series of ligands for the NKG2D activation receptor on natural killer cells and some T cells. NKG2D is a critical receptor for the recognition and elimination of aberrant cells, and its ligands are induced during cellular stress, including infection, malignant transformation or administration of chemotherapeutics [69,95,96]. For example, gemcitabine upregulates the surface protein expression of the NKG2DL, MICA, in pancreatic cancer cells lines, sensitizing them to NK cell killing [67]. Similarly, arsenic trioxide upregulates intracellular and surface protein expression of NKG2DLs (MICA, MICB, ULBP1, ULBP2) in leukemia and breast cancer, possibly through the regulation of heat shock proteins, and sensitizes the cells to NK-mediated destruction [68]. Likewise, 5-fluorouracil time-dependently increases NKG2DL (Mult-1, Rae-1) surface protein on pancreatic cancer cells and acts synergistically with type I IFNα to further induce NKG2DL expression [97]. In this way, chemotherapies can induce broad lymphocyte anti-tumor activity.

As an alternative mechanism of resistance to chemotherapy, emerging evidence suggests that platinum-based chemotherapies may be efficient at targeting differentiated cancer cells, but less effective at eliminating Cancer Initiating Cells (CICs) (Figure 1) [98,99,100]. CICs are a rare population of stem-like cells that possess the capacity to self-renew indefinitely in an undifferentiated state. The canonical Wnt signaling pathway (β-catenin pathway) plays a crucial role in chemo-resistance and the presence of CIC in multiple tumor types, with increased expression of β-catenin correlating with worse prognosis in breast cancer patients [101]. The activation of Wnt pathways augments cell survival, proliferation and cell fate, and accordingly, inhibition of the Wnt/β-catenin signaling pathway can increase sensitivity to chemotherapeutic drugs in various cancer types (Figure 1) [98,99,100]. Wnt/β-catenin signaling is upregulated in platinum-resistant ovarian cancers and was identified as a novel driver of platinum resistance by maintaining cancer cells with stem-like properties [99]. However, targeted inhibition of the Wnt/β-catenin pathway overcame platinum resistance by eradicating CICs [99]. To understand the role of the Wnt pathway in cancer progression, Shah et al. treated human melanoma cell lines and primary melanoma tumors with a recombinant Wnt-3a [101]. While Wnt-3a is thought to function to promote self-renewal of hematopoietic stem cells, neural stem cells and embryonic stem cells, the addition of Wnt-3a to human melanoma cell lines and primary melanoma tumors upregulated the expression of CTLA-4 on the melanoma cells, likely due to CTLA-4 being a direct target of the Wnt/β-catenin signaling pathway (Figure 1) [101]. Further, the Wnt/β-catenin signaling pathway, which is active in ovarian cancer and tumor progression, may regulate the expression of IDO, a known immunosuppressant, by tumor-associated myeloid cells through an LEF-1-associated mechanism [70]. While the source of Wnt ligands within the tumor microenvironment may arise from tumor-associate macrophages or the tumor cells themselves and [70] these studies collectively suggests that Wnt signaling may be operative in contributing to tumor progression by promoting CIC survival and locally regulating immune-suppressive molecule expression, therefore inhibitors that specifically block Wnt signaling may function to reduce cancer cell-mediated immune suppression. However, Wnt signaling also can exert pro-stimulatory immune effects, as described below.

Immune cells that express several Wnt ligands and β-catenin target genes may also exhibit elevated levels of the enzyme Indolamine 2,3-Dioxygenase (IDO) (Figure 1). For example, tumor-associated Dendritic Cells (DCs) display increased levels of IDO and β-catenin target genes, decrease T cell activation in vitro and can induce the differentiation and activation of T regulatory cells (Tregs) in a β-catenin pathway-dependent fashion (Figure 1) [71]. Wnt3a and Wnt5a signaling increase the expression and enzymatic activity of IDO in myeloid DCs in a β-catenin-dependent manner. Wnt5a induces the differentiation of human monocytes into tolerized and immune-suppressive DCs in vitro [71].

## 3. Impact of Radiation and Wnt Signaling on Anti-Tumor Immunity and Cancer Cell Immunogenicity

### 3.1. Radiation Stimulates the Immune System through ICD, Release of TAAs, TLR Engagement and APC Activation

**Figure 2:**
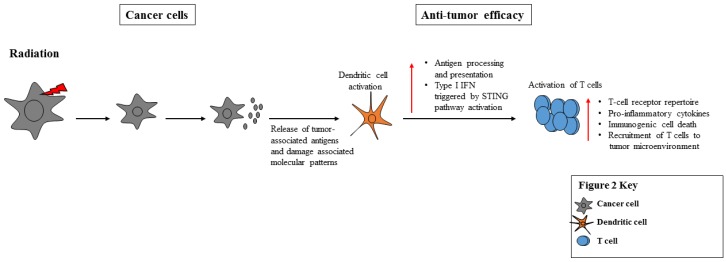
Impact of radiation on cancer cells and anti-tumor efficacy. Radiation is generally an immunostimulatory process that causes immunogenic cell death, inflammatory reactions and recruitment of T cells to the tumor microenvironment. Radiation therapy lyses cancer cells, causing the release of tumor-associated antigens and damage-associated molecular patterns processed and presented by antigen-presenting cells, such as dendritic cells. DCs release type I IFNs in a STING-dependent manner, which can result in activating T cells and eliciting an anti-tumor response.

Radiation can have lymphodepleting effects; however, it also enhances the diversity of the T Cell Receptor (TCR) repertoire of these intratumoral TILs and shapes the repertoire of TIL clones expanded from irradiated lesion [72,102,103]. Upon irradiation, tumor cells release Tumor-Associated Antigens (TAAs) into the environment. The TAAs can then be processed by Antigen-Presenting Cells (APCs), such as Dendritic Cells (DCs), and presented to T cells, resulting in the activated T cells to target the tumor cells that express the TAAs (Figure 2). In this way, TAA-specific T cell responses may be induced in regional lymph nodes and TIL activity locally augmented.

Radiation promotes TAA-specific T cell priming and activation; however, tumor cells themselves have evolved to use various approaches to escape immune cell recognition and destruction. One route of escape from immune cell destruction is by expressing inhibitory molecules, such as CTLA-4 [73,74,104,105,106,107] and PD-L1, on their cell surface, which bind to the respective receptors on the surface of immune cells, prompting an inhibitory immune response, resulting in tumor cell growth [107]. Although CTLA-4 expression is mainly expressed on immune cells, but can also be expressed on tumor cells, such as those found in acute myeloid leukemia, chronic myeloid leukemia, B cell leukemia, melanoma and breast cancers can also express CTLA-4, as well, and the expression of CTLA-4 on tumor cells can trigger apoptosis upon ligand interaction [73,74,104,105,106]. Radiation also induces various immunomodulatory effects, such as the increased expression of pro-inflammatory cytokines IL-1β and TNF-α that play a vital role in the immune system and TIL activation [108].

The observation of the expression of various inhibitory receptors and ligands on immune cells and tumor cells lead to the development of checkpoint blockade antibodies, such as the anti-PD-1 antibody and the anti-CTLA-4 antibody. The anti-CTLA-4 antibody mainly targets the Treg population, hindering their expansion, resulting in the expansion of T cells and an increased CD8^+^/Treg ratio. The addition of PD-L1 blockade reverses T cell exhaustion to mitigate depression in the CD8/Treg ratio, while encouraging oligoclonal T cell expansion.

Ionizing radiation causes immunogenic cell death and inflammatory reactions, in addition to promoting the recruitment of T cells to the tumor microenvironment [102]. One can hypothesize that radiation therapy can transform the tumor into an in situ individualized vaccine, recruiting immune cells to the tumor microenvironment, and the combination of radiation with blocking antibodies against PD-L1 or CTLA-4 can further induce an anti-tumor effect [102]. When mice bearing irradiated or unirradiated tumors were treated with radiation and anti-CTLA-4 antibody, an anti-tumor response was observed, but resistance was detected due to T cell exhaustion and the upregulation of PD-L1 on the melanoma cells [75].

Abscopal effects of localized radiation result when radiation is delivered locally to tumors, but often results in systemic responses at distant tumor sites [109]. The abscopal effects of radiotherapy are believed to be the result of a systemic (radiotherapy-induced) tumor-specific T cell response [109]. Abscopal effects induce endogenous anti-tumor effects in both the innate and adaptive immune system, including tumor recognition and lysis via tumor antigen upregulation and the induction of lymphocyte trafficking into the tumor microenvironment. Radiation therapy facilitates the killing of tumor cells via immunogenic cell death or tolerogenic cell death, but also affords a source of antigen that can be processed and presented by antigen-presenting cells, such as dendritic cells, inducing cross-presentation, and resulting in an antigen-specific anti-tumor response (Figure 2) [109].

### 3.2. Wnt Signaling Activates or Inhibits the Immune System by Regulating the Expression of CD137 or IDO

One component of an effective immune response against tumors involves the trafficking of T cells to the tumor sites and subsequent tumor destruction. However, in order for T cells to effectively eliminate cancer, they must successfully navigate through a complex milieu of stimulatory and inhibitory signals originating from Antigen-Presenting Cells (APCs), malignant tumor cells and the tumor microenvironment, which ultimately determines whether the infiltrating T cells will exhibit potent effector functions against the tumor. Since the presence of T cells infiltrating into the tumor correlates with increased survival in patients with different cancers [78], deciphering the molecular mechanisms involved in T cell infiltration and anti-tumor activity is vital to developing future therapies. Spranger et al. [100] discovered that some T cells are unable to infiltrate into melanoma tumors due to a cancer cell intrinsic oncogenic pathway, involving the Wnt/β catenin pathway. Here, the activation of the Wnt/β catenin pathway through a tumor-intrinsic manner resulted in the inhibition of T cell infiltration into the tumor and resistance to monotherapies using antibodies against PD-L1 and CTLA-4 [100], suggesting that targeted Wnt antagonists, which are entering the clinic, may promote TIL accumulation and response to immune checkpoint blockade.

After initial T cell activation occurs, additional co-stimulatory molecules, such as CD137 (4-1BB), are transiently expressed on immune cells. Although CD137 increases the expansion, effector function and survival of T cells [110,111,112], the role of the β-catenin pathway is only now becoming apparent. Glycogen synthase kinase-3 (GSK-3) is a serine/threonine kinase that is part of the canonical β-catenin/Wnt pathway, which plays key roles in cellular processes involving proliferation, apoptosis and migration [113]. Activation of the CD137 pathway induces ERK, AKT and NFκB signaling pathways and increases TCF1 levels in CD8^+^ T cells [113]. The TCF/LEF transcription factors are the main binding partners and regulatory repressor genes for β-catenin. TCF represses gene expression by binding to the repressor Groucho. The induction of CD8^+^ proliferation by CD137 signaling was dampened when a TCF1/β-catenin inhibitor, quercetin, was used [113]. These results show that CD137 signaling enhances the proliferation of activated CD8^+^ T cells by activating the TCF1/β-catenin axis via the PI3K/AKT/ERK pathway [113]. Gattinoni investigated the role of Wnt/β-catenin signaling on CD8^+^ T cells using a pharmacological inhibitor (TWS119) of the serine/threonine kinase glycogen-synthase-kinase-3β (GSK-3β) [114,115,116]. Inhibiting GSK-3b using TWS119 promoted the accumulation of B-catenin, thus mimicking Wnt signaling. TWS119 also hindered the differentiation of T cells and induced a self-renewing, multi-potent, highly proliferative, with anti-tumor properties Stem Memory T cell (TSCM) subset, ideal for adoptive T cell therapy [114,115,116].

### 3.3. The Impact of Radiation on Cancer Cell Immunogenicity

Radiation enhances the immunogenic cell death of cancer cells leading them to express or release damage-associated molecular patterns (DAMPs), which prompts the stimulation of DCs [117]. In vitro, IFN-β production and DC activation are triggered by tumor cell-derived DNA, via cyclic-GMP-AMP Synthase (cGAS), Stimulator of interferon genes (STING) and Interferon Regulatory Factor 3 (IRF3) (Figure 2). In chemotherapy and radiotherapy, treated cancer cells release ATP and/or High-Mobility Group Protein B1 (HMGB1) and activate DCs via the inflammasome or TLR4 pathways, which in turn contributed to activation of anti-tumor T cells [26,29,118].

The STING complex is a crucial part of the immune-editing process. Cancer cells containing DNA that is damaged can facilitate STING activation. Activation of the STING pathway triggers the production of IFN-β, which alarms the immune system that the body is under attack, inducing the immune system to detect cancerous cells, resulting in activated CD8^+^ T cells, which can migrate to and lyse the tumor cells. STING activation also leads to the production of IL-6, TNF-α and IL-12, as well as upregulated expression of MHCII, CD40 and CD86 by DCs [119]. Overall, the STING pathway plays a crucial role in innate sensing of immunogenic tumors, a process that results in APC activation, IFN-β production and priming of CD8^+^ T cells against tumor antigens. Tumor-derived DNA is likely the ligand for this pathway, which is processed and presented by APCs that can then activate the adaptive immune response [26,29,118].

STING is also required for type I IFN-dependent antitumor effects of radiation. Radiotherapy promotes cell stress and results in the secretion of DAMPs. Radiotherapy enhances the innate immune response in a type I interferon-dependent manner to facilitate the adaptive immune response [120]. For example, radiation increases the production of type I IFNs in DCs. In DCs, STING is required for IFN-β induction in response to irradiated tumor cells. Induction of IFN-β in tumors is diminished in the absence of STING in the host after radiation [121]. Similarly, radiation enhanced robust tumor antigen-specific CD8^+^ T cell responses in wild-type mice; however, antigen-specific CD8^+^ T cell responses in STING-deficient mice after radiation were abolished [122]. Exogenous IFN-β treatment restored CD8^+^ T cell function in STING-deficient mice after radiation, indicating the need for STING-associated type I IFNs in generating robust radiation-induced immune responses against cancer.

The cytosolic DNA sensor cyclic Guanosine Monophosphate-Adenosine Monophosphate GMP-AMP (cGAMP) Synthase (cGAS) mediates the sensing of irradiate tumor cells in DCs. cGAS/STING is a PRR that recognizes pathogenic cytosolic DNA, and DNA from dying tumor cells can be a ligand for cGAS [59,123,124]. In this way, STING/cGAS in DCs is activated by DNA originating from dying cancer cells and critical for high levels of type-I IFN-1 generated during anti-tumor immune response and optimal cross-priming of T cells [59,123,124]. Given the importance of STING/cGAS to the immune response against DNA-damaged cancer cells, STING agonists are now being evaluated. Since the cytosolic DNA-cGAS-STING pathway controls radiation-mediated anti-tumor immunity, the combination of radiation and an STING agonist was tested and found to reduce radioresistance and synergistically increase anti-tumor host response [122]. Moreover, intratumoral injection of STING agonists into mice in the absence of radiation also prompts the regression of established tumors in mice and provides long-lived immunological memory [119].

## 4. Impact of Epigenetic Modifiers on Anti-Tumor Immunity, Cancer Cell Immunogenicity and Inhibitory Ligand Expression

### 4.1. Epigenetic Modifiers Potentiate the Immune Response through TLR Engagement, TAA and Co-Stimulatory Molecules on Immune Cells

**Figure 3:**
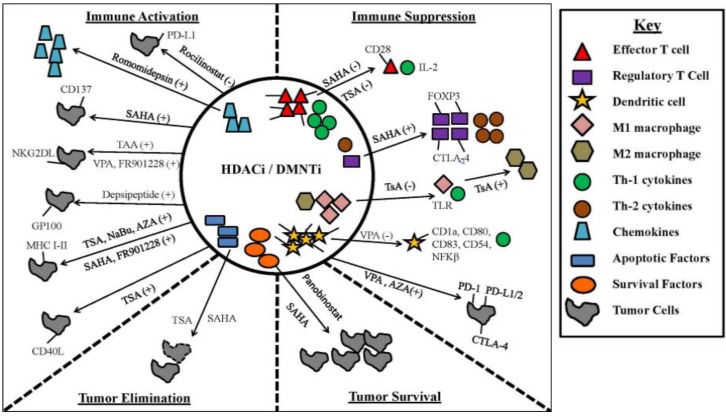
Epigenetic modifiers induce tumor lysis while producing immunostimulatory and immunosuppressive effects on the immune system. Immune system: DC = Dendritic Cell, M1 = M1 Macrophage, M2 = M2 Macrophage, T eff = effector T cell, Treg = regulatory T cell, TLR = Toll-Like Receptor; HDACi: SAHA = Suberoylanilide Hydroxamic Acid (Vorinostat), TsA = Trichostatin A, VPA = Valproic Acid; DMNTi: AZA = 5-Aza-2′-deoxycytidine (Decitabine).

While epigenetic modifiers induce immunogenic cancer cell death and/or cell cycle arrest, they can also enhance or suppress the functions of various cells, including those of the immune system (Figure 3) [125]. In a study conducted by Roger et al., genome-wide expression profiling was used to study the alteration of macrophage Toll Like Receptor (TLR)-induced gene induction by Histone Deacetylases (HDACs) [126]. The HDAC inhibitor Trichostatin A (TsA) inhibited up to 60% of genes that are normally transcriptionally increased by TLR2 or TLR4 stimulation [126]. Specifically, TsA inhibited the following macrophage functions: microbial sensing by Pattern Recognition Receptors (PRRs), signal-transduction mediators, transcription regulators, cytokines, chemokines, growth factors and co-stimulatory molecules [126]. HDACi can also affect other immune cells, in addition to macrophages. For example, HDACi TsA or suberanilohydroxamic acid (SAHA) can induce thymic production of murine FoxP3^+^ Tregs, stimulate the conversion of peripheral T cells into Tregs and enhance Treg suppressive function in vitro and in vivo [127,128]. Interestingly, Akimova et al. showed that the augmented suppressive function of HDACi-exposed Tregs was associated with their increased expression of CTLA-4 [128]. In addition to the effects on Tregs, in vivo use of HDACi (TsA or SAHA) can inhibit effector T cell cytokine production and proliferation and promote T cell anergy [129,130,131], and treatment with TsA also abrogates IL-2 production and CD28 expression by CD4^+^ T cells [129,130,131].

HDACi exposure can also impact the innate arm of the immune response. For instance, treatment of DCs with HDACi, Valproic Acid (VPA) and MS-275 diminishes their differentiation and function, demonstrated by the downregulation of co-stimulatory markers CD1a, CD80 and CD83 and adhesion marker CD54 [132,133]. HDACi therapy is known to impair the activation of NFκB in DCs and macrophages and to impair their maturation and differentiation [134]. HDACi can also halt the production of multiple pro-inflammatory cytokines in APCs and induce the conversion of anti-tumor macrophages (M1) into pro-tumor M2 cells [127,128].

The expression of chemokines in cancer cells, T cells and macrophages can also be increased by HDACi (Figure 3). Specifically, the HDACi romidepsin induces the expression of T cell chemokines in cancer cells and increases T cell infiltration into the tumor and T-cell mediated tumor regression [135,136,137]. HDACi (SAHA, Vorinostat) possesses anti-tumor effects in in vitro and in vivo settings [63,94,138]. SAHA induces sensitization of cell death receptor-resistant breast cancer cells to cell death [139,140,141]. Bellarosa and colleagues found that the co-stimulatory molecule, CD137, is augmented by SAHA treatment in breast cancer cells [142]. Here, the upregulation of the CD137 receptor/ligand pathway correlated with a synergistic cytolytic effect when MDA-MB-231 cells were treated with the combination of SAHA and soluble CD137 [142]. This finding could indicate novel combination treatment using SAHA with CD137 agonistic antibodies for the treatment of breast cancer tumors.

In NK cells and CD8^+^ T lymphocytes, NKG2D activation triggers cytotoxic effector function. A number of immune cell-based therapies depend on recognition of NKG2DLs, such as MICA and MICB in humans, on tumor cells for targeting. One way in which tumors escape immune cell recognition is by downregulating the ligands; however, HDACi can upregulate the cell surface expression of MICA/B in certain tumors [143]. Pre-screening of the expression of cell surface NKG2DLs on tumors could assist in determining who may benefit from the HDACi prior to immune cell-based therapies utilizing the NKG2D pathway. Although HDACi can induce immunosuppressive effects, HDACi (TAA and VPA) increases the expression of NKG2D ligands (NKG2DL) on various tumor types [144,145], which triggers NK and CD8^+^ T cell cytolytic activity against tumors [144,145]. HDACi VPA increases the expression of MICA, MICB and ULLBPs in human hepatocellular carcinoma cells resulting in enhanced recognition and killing by NK cells in vitro [146,147,148]. Indeed, cancer cells exposed to HDACi exhibit an upregulation of NK cell-activating ligands, MHC class I and II molecules, co-stimulatory molecules, as well as components of the machinery required for antigen presentation [56,144,145,149,150]. Furthermore, the HDACi depsipeptide can also enhance the expression of tumor antigen gp100 in murine melanoma cells, resulting in improved recognition by gp100-specific T cells [148]. Wargo demonstrated that the demethylating agent 5-Aza-2′-deoxycytidine used alone or in combination with depsipeptide increased the expression of tumor antigen NY-ESO-1 on tumor cells, resulting in increased IFN-γ responses by antigen specific (NY-ESO-1^+^) T cells [64].

### 4.2. Epigenetic Modifiers Induce Tumor Expression of Inhibitory Immune Checkpoint Molecules

Like conventional chemotherapeutics, HDACi amplified ICD. In colon carcinoma cell lines, administration of the HDACi Vorinostat promoted tumor cell engulfment by dendritic cells and facilitated the expression of surface CRT and the release of HMGB1 and ATP [65,138,151]. However, to date, there are no in vivo models demonstrating “bona fide” ICD induction by HDACi or DNA methyltransferase inhibitor (DNMTi). However, several animal studies suggest that induction of ICD (or at least components of ICD) may be elicited by HDACi and DNMT. For example, co-administration of the HDAC inhibitor Valproic Acid (VPA) or Suberoylanilide Hydroxamic Acid (SAHA) with AZA synergistically acts to upregulate the expression of Tumor-Associated Antigens (TAA) in a mouse model of mesothelioma, leading to CD8^+^ T lymphocyte infiltration and IFNγ secretion, possibly the result of ICD [152]. Likewise, Photodynamic Therapy (PDT) can induce ICD, and simultaneous treatment with PDT and AZA results in potentiated antitumor effects and CD8^+^ T cell activation in lung, mammary and colon carcinomas, through enhanced antigen expression and enhanced ICD [153,154,155]. Hence, epigenetic modifiers may act alone or concomitantly with conventional chemotherapeutics to promote immunogenic cell death. Notably, several chemotherapeutic agents do not bring about ICD, at least by the currently-accepted definition, although they may meet one or more requirements [125]. In summary, cumulative evidence from preclinical studies suggests that agents that stimulate ER stress, autophagy, CRT translocation, ATP/HMGB1 release and type I IFN secretion can enhance the efficacy of standard chemotherapeutic regimens by enhancing immunogenicity, reestablishing immune surveillance and promoting an antitumor response.

HDACi also upregulates MHC classes I and II by cancer cells (Figure 3). Vorinostat, an HDACi used in clinical trials to treat acute myeloid leukemia, increased surface levels of MHC classes I and II in lymphoma by activating IFNγ signal transduction via the IFNγ receptor 1 [138,150]. Similarly, the HDACi, FR901228, facilitated a time- and concentration-dependent increase in HLA-A/B surface/intracellular protein and RNA expression in leukemia and increased HLA-A/B surface expression in hematological and colon malignancies by potentiating the activity of glycogen synthase kinase (GSK-3β) [56]. Trichostatin A (TSA), a class I and II HDACi, increased the expression of MHC class I and II surface protein and CD40 ligand expression in melanoma [156]. Similarly, TSA elevated MHC class II protein and RNA (HLA-DR) transcription in neuroblastoma and increased MHC class I protein, RNA transcription and CD40 ligand (CD40L) expression in both lymphoma and colon cancer [149]. Notably, CD40 is a co-stimulatory receptor located on antigen-presenting cells that is required for their activation. Binding of CD40 on CD4^+^ T cells to its respective ligand, CD40L, found on tumors leads to cytotoxic T cell recruitment and elicits an anti-tumor effector response [157].

Like traditional chemotherapeutics, DNMTi can induce the expression of immunosuppressive ligands. Decitabine, a hypomethylating agent, upregulated PD-1, PD-L1, PD-L2 and CTLA-4 protein and RNA transcription in leukemia patients and dose-dependently increased these same ligands in leukemia cell lines [158]. In addition, Azacitidine (AZA) elevated PD-L1 surface expression and RNA transcription in lung carcinoma through activation of STAT transcription and anti-viral defense signaling [159]. Likewise, AZA appears to augment CTLA-4 expression in a murine model of melanoma through similar mechanisms [160]. Like PD-L1/2, CTLA-4 transmits inhibitory signals to T cells, suppressing T cell activation and thus hindering the immune response [161,162].

Histone deacetylase inhibitors, like DMNTi, produce anti-tumor effects by changing the expression levels of oncogenes or tumor suppressors or through modifying the acetylation/deacetylation of histones and/or non-histone proteins, such as transcription factors [163]. Not surprisingly, HDACi can modify the expression of inhibitory molecules (Figure 3) [44]. For example, class I HDACi were demonstrated to upregulate PD-L1/L2 surface protein and RNA transcription in melanoma patients, in melanoma cell lines and in a syngeneic mouse model of melanoma, through acetylation of the PD-L1 and PD-L2 promotor [164]. In our laboratory, administration of Valproic Acid, a class I and II HDACi, potently upregulated PD-L1 surface protein expression and RNA transcription in a time- and concentration-dependent fashion in ovarian cancer, via a mechanism that likely involves STAT signaling [51].

The combination of HDACi with checkpoint blockades has been investigated. Using a lung cancer model, the combination of HDACi romidepsin and anti-PD-1 blocking antibody significantly increased the response to the anti-PD-1 blocking antibody, while enhancing the cytolytic function, chemokine expression and activation of TILs [135,136,137]. In a melanoma model, class I HDACi upregulated the expression of PD-L1 and to a lesser extent, PD-L2 [164]. Specifically, the HDACi treatment induced rapid upregulation of histone acetylation of the PD-L1 gene [164]. Mice containing melanoma B16 tumors received a combination of HDACi and PD-1 blockade [164]. This combination resulted in slower tumor progression and overall increased survival, compared with monotherapy and control groups [164].

Importantly, not all chemotherapies or epigenetic anticancer agents enhanced inhibitory ligand expression (Table 2). For example, doxorubicin (and to a lesser extent, daunorubicin) downregulated PD-L1 cell surface/cytosolic protein expression on breast cancer cells in vitro and in vivo, while upregulating nuclear expression through PI3K/AKT and non-PI3K/AKT mechanisms [165]. The same group also reported that cisplatin, docetaxel and mitoxantrone did not change on PD-L1 surface ligand expression in breast cancer cell lines [165]. Likewise, cisplatin was shown to inhibit PD-L2 surface protein expression in melanoma cells through suppression of STAT6 phosphorylation [166]. Similarly, the HDAC6 inhibitor, Rocilinostat, suppressed PD-L1 expression in B cell leukemia cells and in primary B cells isolated from CLL patients [167]. In addition, AZA did not alter PD-L2 surface expression nor RNA transcription in lung carcinoma [159]. Because particular chemotherapies may regulate inhibitory ligand expression through distinct pathways that may be time-, concentration- and tumor-specific, additional studies are required to better understand the mechanisms of chemotherapy-induced PD-L1/L2 and CTLA-4 expression in aberrant cells and its potential relevance to adjuvant antibody and immune therapies.

The Cancer Genome Atlas (TCGA) project revealed that more than 10,000 human tumors contained defects in DNA methylation [168]. DNA methylation acts to repress gene transcription and is involved in the regulation of human leukocyte antigens, and MHC class I downregulation in cancer is associated with promoter hypermethylation [48,169]. Hence, administration of DNMTi can reverse chronic MHC class I suppression found in many malignancies [44]. Azacitidine, for example, potentiated MHC class I surface expression in vitro and in vivo in murine lung carcinoma by increasing the transcription of antigen-presenting genes including TAP-1, TAP-2, LMP-2, LMP-7 and tapasin [170,171,172]. Likewise, AZA increased HLA class I surface expression and RNA transcription in human melanoma cell lines [173,174] and can thus be used to sensitize cancer cells to T cell-mediated attack.

## 5. Conclusions

**Figure 4:**
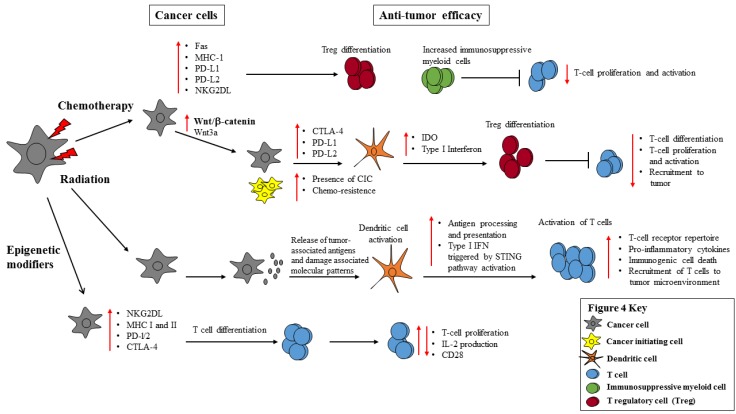
The impact of chemotherapy, radiation and epigenetic modifiers in cancer cell expression of immune inhibitory and stimulatory molecules and anti-tumor efficacy. Genomic destabilizers (chemotherapy and radiation) and epigenetic modifiers elicit immunogenic and non-immunogenic cell death of cancer cells and induce the expression of immune stimulatory ligands. Conversely, genomic destabilizers and epigenetic modifiers can also upregulate inhibitory ligands and pathways and promote the differentiation of suppressive cells, prompting the suppression of the immune system.

Genomic destabilizers, such as radiation, chemotherapy and epigenetic modifiers, are standard and emerging therapies for the treatment of cancer. In addition to producing DNA damage (or immunogenic cell death), these therapies can also have diverse effects on the anti-tumor efficacy of the immune system and alter the tumor microenvironment (Figure 4).

Chemotherapy, for example, not only elicits immunogenic cell death, but also upregulates the expression of inhibitory ligands. Inhibitory ligands, such as PD-L1, PD-L2 and CTLA-4, on cancer cells inhibit T cell proliferation and activation. This may result in escape from host anti-tumor immunity and unimpeded proliferation. Conversely, radiation is generally an immunostimulatory process that causes immunogenic cell death, inflammatory reactions and recruitment of T cells to the tumor microenvironment. In addition, radiation therapy lyses cancer cells, causing the release of tumor-associated antigens, which can be processed by antigen-presenting cells, such as dendritic cells. The dendritic cells then present the antigens to T cells, activating the T cells and eliciting an anti-tumor response. In addition, epigenetic modifiers eradicate cancer cells, increase NK and T cell pro-stimulatory ligands, but induce the conversion of T cells into T regulatory cells. T regulatory cells suppress T cell activation and proliferation, allowing the cancer cells to multiply and evade cell death. Although epigenetic modifiers increase inhibitory ligands on cancer cells, they can increase HLA class I and II expression and be immunostimulating depending on the malignancy.

Further, immunotherapeutic antibodies that target the inhibitory ligands expressed on cancer cells have been developed. However, utilization of these antibodies as a single agent is often limited to a subset of patients, and response rates are relatively modest in most diseases, especially solid tumors that are heterogeneous or naturally low in expression. Nevertheless, combinatory immunotherapy strategies, including multiple checkpoint blockade, adoptive T cell therapy, immune-based vaccines and cytokine therapies, in conjunction with genomic destabilizers should be considered. In addition, the use of different and potentially concurrent therapies will require careful consideration of the timing and dosing of the dissimilar reagents, the importance of the receptor/ligand expression on the target cells and the mechanisms affecting expression. In addition, depending on the tumor microenvironment and expression of inhibitory receptors and ligands on the immune cell and tumor cell surface, specific combinations may not to be universal, and integrative treatments may need to be tailored to each, individual patient.

## Figures and Tables

**Table 1:** The effects of chemotherapy, lymphodepletion and radiation induce immunogenic cell death, antigen presentation and enhance T cell priming and activation. Und = Undetermined Observations.

**I. Examples of Agents Which Induce ICD**	**Cancers**	**Triggers**	**CRT**	**ATP**	**HMGb1**	**Type I IFNs**	**ICD**	**Immune Impact**	**Ref.**
Anthracyclines	Breast, Colon, Sarcoma, Leukemia	ER Stress/Autophagy						CD8^+^ T activation, proinflammatory	
i. Doxorubicin			Yes	Yes	Yes	Yes	Yes	cytokine release, secondary necrosis	[20,21,22,23,24,26,27,28,29]
ii. Epirubicin			Yes	Yes	Yes	Und	Yes	likely enhancing antigen presentation	[21,22,23,24,29]
iii. Idarubicin			Yes	Und	Yes	Und	Yes		[20,29]
iv. Mitoxantrone			Yes	Und	Yes	Und	Yes		[20,22,23,27,29,30,31]
Bortezomib	Lymphoma, Myeloma	ER Stress, HSP Exposure	Yes	Und	Yes	Yes	Yes	DC maturation/activation	[34,35,36,37]
Bleomycin	Colon	ER Stress/Autophagy	Yes	Yes	Yes	Yes	Yes	CD8^+^ T activation, and proinflammatory	[23,32,33,39]
								cytokine release	
Cisplatin	Colon	-	No	Und	Yes	Und	No	Limited	[22,40]
Cyclophosphamide	Lymphoma, Glioma	ER Stress	Yes	Yes	Yes	Yes	Yes	CD8^+^ T, NK, macrophage activation	[22,25,27,30,40,53]
Oxaliplatin	Colon, Mouse sarcoma	ER Stress/ Autophagy	Yes	Und	Yes	Und	Possible	TLR4 engagement, DC activation	
Vorinostat (HDACi)	Colon, Central Nervous System		Yes	Yes	Yes	Und	Likely	DC, B cell activation	[63,64,65]
**IIa. Examples of Agents Which Do/Do Not Enhance MHC (Antigen Presentation)**	**Cancers**	**Increased HLA Expression**	**Cytokine Effects**						
Carboplatin (Cisplatin)	Ovarian, Lung	No (Ovarian); Yes (Lung)							[51,66]
Cyclophosphamide	Kidney, Breast, Prostate, Colon	Yes							[52]
Gemcitabine	Ovarian, Breast, Kidney, Prostate, Colon	Yes							[52]
HDACi (FR901228)	Leukemia, Lymphoma, Breast, Cervical	No							[13]
Interferons (Type I or II)	Ovarian, Melanoma	Yes							[50,51]
Microtubule destabilizers	Ovarian		Increased IFNα						
i. Epothilone B		Yes	IL-1β, IL-6, IL-12						[48]
ii. Taxol		Yes							[48]
iii. Vinblastine		Yes							[48]
Oxaliplatin	Kidney, Breast, Prostate, Colon	Yes							[52]
Paxclitaxel or Paxclitaxel-Carboplatin	Ovarian	Yes							[42]
**IIb. Examples of Agents Which Enhance NKG2D (Antigen Presentation)**	**Cancers**	**Immune Impact**						**Specific NKG2D Ligand**	
Arsenic Trioxide	Leukemia, Breast	NK/HSP activation						MICA, MICB, ULBP1/2	[67]
5′-Flurouracil	Pancreas	Synergy with Type I IFNs						Mult-1, Rae-1	[68]
Gemcitabine	Pancreas	NK activation						MICA	[69]
**III. Lymphodepleting Agents Improve T Cell Function (T cell Activation, Persistence)**	**Cancers**								
Cyclophosphamide	Melanoma, Several Others	Increased Type I IFNs, TLR/DC activation, Treg depletion, increased Th17 cells, TRAIL activation, improved persistence of administered T cells							[23,54]
Fludarabine	Melanoma	Increased IL-7, IL-15, improved persistence of administered T cells							[61]
Irradiation	Melanoma	Increased IL-7, IL-15, homeostatic space and persistence of administered T cells, Treg depletion, release of immunostimulatory gut microflora							[10,60]
**IV. Radiation as a Immunostimulatory Treatment (Antigen release, presentation, T cell/ and APC Priming)**	**Multiple Cancers**	**Increased Release of TAAs, improved antigen processing/presentation**							[70,71,72,73,74,75]
		Proinflammatory cytokine secretion							
		ICD							
		Recruitment of immune cells to tumor microenvironment							

**Table 2:** Chemopreventative Agents Alter the Expresson of Immunosuppressive Ligands (PD-L1 and PD-L2).

Chemotherapeutic	Category	Tumor Type	PD-L1 Protein	PD-L1 RNA	In Vivo	PD-L2 Protein	PD-L2 RNA	Mechanism	Ref.
Carboplatin	Alkylating Agent	Ovarian	+ (S/I)	+	+ (M)	OBS	OBS	NFKb	[2]
Carboplatin	Alkylating Agent	Ovarian	+ (S)	+	+ (M)	+ (S)	+	JAK/STAT, Antiviral Defense	[51]
Cisplatin	Alkylating Agent	Liver	+ (S)	OBS	OBS	OBS	OBS	MEK-ERK-MAPK	[94]
Cisplatin	Alkylating Agent	Breast	NC	OBS	OBS	OBS	OBS	-	[28]
Cisplatin	Alkylating Agent	Melanoma	− (S)	OBS	OBS	OBS	OBS	STAT6 Inhibition	[28]
Docetaxel	Alkylating Agent	Breast	NC	OBS	OBS	OBS	OBS	-	[28]
Gemcitabine	Antimetabolite	Ovarian	+ (S/I)	+	+ (M)	OBS	OBS	NFKb	[2]
5-Fluorouracil	Antimetabolite	Breast	+ (S)	OBS	OBS	OBS	OBS	JAK/STAT, MAPK, PI3K/AKT	[4]
Paclitaxel	Antimicrotubule	Breast	+ (S)	OBS	OBS	OBS	OBS	JAK/STAT, MAPK, PI3K/AKT	[4]
Paclitaxel	Antimicrotubule	Ovarian	+ (S/I)	+	+ (M)	OBS	OBS	NFKb	[2]
Paclitaxel	Antimicrotubule	Colon	+ (S/I)	+	OBS	OBS	OBS	MEK-ERK-MAPK	[6]
Paclitaxel	Antimicrotubule	Liver	+ (S/I)	+	OBS	OBS	OBS	MEK-ERK-MAPK	[6]
Azacytidine ^a^	DMNTi	Lung	+ (S)	+ (S)	OBS	NC	NC	STAT, Antiviral Defense	[17]
Decitabine ^a^	DNMTi	Leukemia	+ (S/I)	+	+ (P)	+ (S/I)	+	NE	[16]
HDACi (s)	HDACi Class I	Melanoma	+ (S)	+	+ (P/M)	+ (S)	+	Acetylation of PD-L1/2 Promotor	[27]
Valproic Acid	HDACi Class I, II	Ovarian	+ (S)	+	OBS	OBS	OBS	JAK/STAT	[51]
Rocilinostat	HDACi Class VI	Leukemia	− (S)	OBS	− (P)	OBS	OBS	NE	[30]
Doxorubicin	Topoisomerase (−)	Breast	− (S/I) + (N)	OBS	+/−	OBS	OBS	PI3K/AKT, non-PI3K/AKT	[28]
Etoposide	Topoisomerase (−)	Breast	+ (S)	OBS	OBS	OBS	OBS	JAK/STAT, MAPK, PI3K/AKT	[4]
Etoposide	Topoisomerase (−)	Occular	+ (S/I)	+	OBS	OBS	OBS	miR	[8]
Mitoxantrone	Topoisomerase (−)	Breast	NC	OBS	OBS	OBS	OBS	-	[28]
Trabectedin	Undefined Cytoxin	Ovarian	+ (S/I)	OBS	+ (M)	OBS	OBS	IFNg release	[5]
Arsenic Trioxide	Undefined Cytoxin	Leukemia	+ (S/I)	OBS	OBS	OBS	OBS	miR	[9]

+ = increase; − = decrease; S = surface; I = intracellular; N = nuclear; NC = no change; NE = not evaluated; M = mouse; P = patient; OBS = unpublished observation; a = also induces CTLA-4.

## Abbreviations

The following abbreviations are used in this manuscript:

ACT	Adoptive Cell Transfer
APCs	Antigen-Presenting Cells
AZA	Azacitidine
cGAS	Cyclic-GMP-AMP Synthase
CICs	Cancer-Initiating Cells
CRT	Calreticulin
DAMPs	Damage-Associated Molecular Patterns
DC	Dendritic Cell
DNMTi	DNA Methyltransferase Inhibitor
GSK-3	Glycogen Synthase Kinase-3
HDACs	Histone Deacetylases
HDACi	Histone Deacetylase Inhibitor
HMGB1	High-Mobility Group Protein B1
HSP	Heat Shock Proteins
ICD	Immunogenic Cell Death
IDO	Indolamine 2,3-Dioxygenase
IRF3	Interferon Regulatory Factor 3
MSI	Microsatellite Instability
PLD	PEGylated Liposomal Doxorubicin
PRRs	Pattern Recognition Receptors
SAHA	Suberanilohydroxamic Acid
STING	Stimulator of Interferon Genes
TAA	Tumor-Associated Antigens
TCGA	The Cancer Genome Atlas
TCR	T Cell Receptor
TIL	Tumor-Infiltrating Lymphocytes
TLR	Toll-Like Receptor
Tregs	T Regulatory Cells
TsA	Trichostatin A
TSCM	Stem Memory T Cell
VPA	Valproic Acid
